# Hospital and Institutional News

**Published:** 1915-02-06

**Authors:** 


### HOSPITAL AND INSTITUTIONAL NEWS.

### THE POOR-LAW CONFERENCE.

So many questions of vital importance to Poor-Law administrators are found to arise during the continuance of the war that the decision to hold the Central Poor-Law Conference as usual this year was undoubtedly a wise one. The conference is to meet on Tuesday and Wednesday of next week, and additional interest will be lent to the proceedings by the fact that Mr. Herbert Samuel, President of the Local Government Board, is to preside over the meeting and will deliver an address on Tuesday morning. After Mr. Samuel's address, papers on "The Relief of Distress during War Time" will be read by Mr. Gladstone Walker, Clerk to the Guardians of Newcastle-on-Tyne, and by Mrs. Radiham (Cambridge). Tuesday afternoon will be devoted to a general discussion of these papers. On Wednesday morning Mrs. Mylne, late Chairman of the Paddington Board of Guardians, will read a paper on "The Local Government Board Circular on Relief to Widows and Children," and in the afternoon the Rev. G. A. Suttle, late Chairman of the West Ham Board of Guardians, will initiate a discussion on "The Poor-Law in Relation to the Blind and other Physical Defectives."

### SHOULD CONFERENCE DELEGATES BE PAID THEIR EXPENSES?

An important question of principle arose at a recent meeting of the Kidderminster Board of Guardians out of the apparently minor matter of whether the expenses of a Guardian and the clerk in attending the forthcoming Poor-Law Conference in London should be paid. The Rev. W. Wykes-Finch violently opposed the proposal on the ground that they had been establishing a principle, with which he did not agree, of paying the expenses of a clerk and others to conferences. He further remarked that there was a growing tendency on the part of the Local Government Board to override the Guardians and to centralise everything, towards which conferences acted as an encouragement. Up till to-day delegates had attended the conferences at their own expense. It was decided not to send the delegates. Mr. Wykes-Finch adopts two lines of argument which should be distinguished from each other. In the first place he condemns conferences because in his opinion they increase an existing centralising tendency; and, secondly, he declares that hitherto delegates have paid their own expenses. As to the first, a statesmanlike view of the situation reveals that more centralisation is what is wanted, if existing Poor-Law institutions are to be made full use of, since a properly classified arrangement of patients only centralisation can secure. The second matter, the expenses of delegates, is one on which opinions may more fairly differ. Some authorities make a custom of paying the expenses of delegates, and others decline to do so. That the conferences themselves are valuable, however cumbersome is the machinery, cannot be denied. They stimulate thought and activity, and make a channel of inter-communication for all the centres represented at them. It is just this stimulus, apparently, that Mr. Wykes-Finch fears; but on its presence depends the development of Poor-Law officers' powers and usefulness.

### THE HAMPSTEAD APPOINTMENT.

In view of the correspondence which we print elsewhere in this issue on the above subject it is interesting to give what details are available concerning the career and general experience of Mr. J. A. Kyle, the new secretary of Hampstead General Hospital. He resided at Balliol House, attending the classes given for students at Toynbee Hall, and is understood to have started life as an engineer and to have had experience of accountancy. Two years ago he was a candidate for the post of steward at the Royal Hospital for Incurables. As we noted last week, Mr. Kyle was Sir Robert Baden-Powell's right-hand man in the organisation of the Boy Scout movement, and in this connection his athletic tastes deserve mention.

### DOCTORS' COMMISSIONS AND POSTS KEPT OPEN.

Now that the continuance of the war has familiarised the various authorities with the difficulty of replacing medical volunteers for active service, less readiness is shown to offer to keep open the places of those who apply for permission to serve. For instance, after a lengthy discussion, the primary sub-committee appointed by the Wolverhampton Education Committee to consider the application of Dr. Spencer Badger, school medical officer, to join the Forces, has reported that "as it would be impossible satisfactorily to proceed under the advice of a *locum tenens* with the school medical service and the establishment of a school clinic," if the doctor accepts a commission it will determine his engagement. We can sympathise with both sides here, and as the chairman of the Education Committee recently remarked, for the developments they have in view the guidance of their school medical officer was essential. We are glad also to see that the attempt made to interpret the committee's attitude as that of prevention of the doctor from serving his country was not successful. It is perhaps worth while remarking that, though doctors are free from the danger, the general vague promise to keep open places for all and sundry must break down in many cases, as it did after the South African War, since a campaign of months or years may easily make a man unfit to resume, say, office duties or to serve in a shop. Doctors who go to the Front, of course, enlarge their experience and continue their profession, but that does not alter the fact that they are wanted also at home, and that it may be more patriotic and useful sometimes to stay there.

### REMARKABLE HOSPITAL CHAPELS.

In the series of illustrated articles on "Provincial Hospital Chapels" now appearing in The Hospital, our readers will not fail to note how in each case some individual feature—whether in the decoration of the chapel or the names memorialised on its walls, or in the hymns which the chaplain has written for its special use—exists of general as well as personal interest. We believe that there is no hospital chapel which has not some association of this kind which may prove an inspiration to others, and therefore ask the many hospital chaplains and other officials whose chapels are not mentioned here to follow the example of the writer and send us, with photographs, those features of their chapels which stand out and differentiate each from its fellows. Having in the present series of articles followed up accounts of some noteworthy hospital chapels in London, we would like to make the series complete, which can be done by the co-operation of our readers. Nothing stimulates *esprit de corps* and institutional patriotism more than the record of individual features of the kind disclosed in these articles. Names and achievements take on a new life when presented for the consideration of those outside the institution which possesses them; and no one will fail to take an enhanced interest in the chapel where they attend who sees its individual points of interest and difference set out in an illustrated record among the chapels of provincial hospitals. We therefore invite particulars from our readers for publication in THE HOSPITAL. We hope to publish a further instalment of the series next week.

### THE SCARCITY OF SCIENTIFIC GLASSWARE.

SINCE the war began difficulty has been experienced in obtaining various classes of glass apparatus used in analytical laboratories and technical schools, as well as in the scientific departments of hospital dispensaries. Fortunately, however, as the result of the perseverance of English glassmakers, the inconvenience is steadily diminishing, and it is hoped that before very long the difficulty will disappear altogether. But for the present it is still necessary to use scientific glassware economically, and especially to avoid breakages as far as possible. The kind of glassware that is most precious just now is that made of "Jena" glass, which before the war used to be imported from Germany in sufficient quantity. In some of the special operations of analysts and research chemists this glass is no doubt the best, but for most ordinary purposes apparatus made from varieties of glass that are more readily obtainable is quite suitable, and should therefore be used. If this is done, the comparatively small quantity of Jena glassware available could be earmarked for use in those manufacturing industries, including some of special value in the present crisis, which require this special glassware for purposes connected with various industrial processes. The Board of Education has invited education authorities and governors of schools and colleges to assist in this matter by being very sparing in the use of German and Austrian glassware. The Board has, in fact, asked that stocks should be examined and a careful record kept of quantities and consumption, and has recommended that fresh orders should not be given to supplying agencies for the present where this can possibly be avoided. No doubt hospitals and medical schools in which glassware of the kind in question may be occasionally used will be desirous of co-operating with the Government in this matter.

### MOTOR BACTERIOLOGICAL LABORATORY FOR THE WELSH ARMY CORPS.

MRS. LYNN THOMAS, wife of the Cardiff surgeon, Mr. Lynn Thomas, C.B., has presented to the Welsh Army Corps, of which her husband is medical director, a fully equipped motor bacteriological laboratory costing about £1,500. This will be attached to the 1st Division of the Corps, and is similar to those found on the Continent. Mr. Lynn Thomas has made a deep study of the action of bacteria during warfare, and has particularly acquainted himself with the work accomplished by the Japanese during their war with Russia. His wife's gift will be valuable to the Welsh Medical Service which he is organising.

### A COTTAGE HOSPITAL FOR COALVILLE.

THE Leicestershire town of Coalville, the centre of a large mining population, is likely soon to establish a cottage hospital. The originator of the movement is Mr. John Wootton, J.P., a local resident, who, at a meeting convened for the purpose, indicated his desire to see a cottage hospital established in the town in memory of his wife and son, and made an offer of £1,000 for the purpose. The offer was accepted with gratitude, and as Mr. Wootton wishes the hospital to be completed in six months, it may be inferred that no time will be lost in appointing a committee to give full consideration to the many details arising, and to the best means of raising money necessary for maintenance. The drawback to new movements on a small scale generally is that they start with enthusiasm, but in the end raise money for maintenance at the expense of existing institutions. We hope that the Coalville committee will guard against this, and make the hospital, as it ought to be, a Coalville institution supported entirely by the district which it is to serve.

### THE ART OF COLLECTING.

As an instance of the success which may attend hospital collections at the present time, notwithstanding the huge amount which the charitable public has subscribed for other purposes, we may record the total raised on behalf of the Gravesend Hospital in connection with the Christmas gift collection. This annual effort actually produced £100 more than was subscribed last year, an achievement on which Mr. Walter Pearson, the secretary, and his band of collectors may be congratulated. The grand total, after the deduction of incidental expenses, was £275. The system on which the collection is worked is that of districts, into eighty-three of which the hospital area was divided, and the sums collected varied from under a sovereign per collector to the two easily most successful sums of £14 12s. through Mrs. Wade and Mrs. Dines, and £13 11s. through Mrs. T. H. Dunch. The proportion of houses responding to those visited appears from a glance at the figures to have varied considerably, but where, as in the two cases above quoted, (over) 50 per cent. did so a satisfactory result was generally secured. All in all, rather less than half the houses visited subscribed something. The average sum obtained by each collector was £2 8s. 10d., and their object, it must be remembered, is to get a Christmas present for the hospital.

### THE HEIGHT OF THE STAFF.

Next Tuesday evening Dr. M. S. Pembrey is to remind an audience of how largely ornamental has been an army's function in the past by opening a discussion at the Royal Sanitary Institute on "Tall *versus* Short Men for the Army." We are rather surprised that there is supposed to be any doubt on the matter, so obviously is the height, which is becoming in a footman, out of place and dangerous in a soldier. By a coincidence, simultaneously with the announcement of the discussion, Lord Rosebery, that apt reflector of the forces of the hour, was speaking in support of a Bantam Battalion in Edinburgh. He expressed himself strongly that a 5-foot man was just as good as a 6-foot man for any practical purpose. For military purposes he had no doubt they fought and shot just as well; they were more compact. They were, he added, apt to have stronger constitutions, were lighter on a horse, and offered a much smaller mark. He defied any tall man to contradict him! We only hope that Lord Rosebery will rise from the body of the hall on Tuesday night and emphasise these facts, if there is any academic attempt to minimise them. Surgeon-General Sir W. Launcelot Gubbins will preside. The experience of hospital officers with porters and nurses makes them realise the value of the 5-foot physique.

### ORGANISING AN APPEAL DEPARTMENT.

Very interesting reading can be provided by such documents as appeal committees' reports, and this is certainly the case with that presented last week to the court of St. Bartholomew's Hospital. During the year 1914 the appeal committee remark that 20,000 letters were sent out to a selected list of likely donors. The actual total received during the three years that the special appeal was being carried on was in round figures £53,000, and this in spite of the fact that appeals on behalf of the *Titanic* and the quinquennial appeal of the London Hospital were promulgated at the same time. During 1914 itself the total received was £10,236 from 1,294 subscribers and donors, divided roughly into equal sums from an equal number in each class. The year's expenses, including the salaries of the appeal clerk and temporary assistants, together with the £285 spent on printing and stationery and £130 on postages, amounted to £722, or 7 per cent. on the sum collected. Moreover, it must be remembered that the committee's activities also reap a reward in legacies and large grants, which are directly or indirectly influenced by such propaganda. The committee, then, has accomplished its aims, the £50,000 loan from the bank has been paid off, the appeal department has now been established on a permanent basis, and subscribers have been gained for the first time contributing over £5,000 per annum, largely thanks to the time and hard work which Mr. Edwin Layton has devoted to it in his capacity of honorary secretary.

### COUNTY RADIUM INSTITUTES.

Some interesting facts with regard to the *x*-ray installation were discussed at the recent annual meeting of the Newton Abbot Hospital and Dispensary. The *x*-ray installation has been enlarged at a cost of £100, and the appeal for money to purchase radium for the hospital brought in a sum of £3,586, of which two-thirds have been spent at present. In the meantime twenty cases have been treated by its means in the hospital, and forty-eight separate applications have been made. The report, which was presented by Mr. H. H. Rickard, R.N., the honorary secretary, states that in five or six cases the results were good. But from an administrative point of view the possession of this new department is causing the hospital's committee some anxiety. While the hospital naturally is intended to serve the district in which it is placed, its new radium department has led to applications for admission being received from remote parts of Devonshire. Indeed, the question of building cancer wards in the hospital grounds has only been postponed until the end of the war, and then it is possible that a movement to provide a county radium institute, already discussed, may be adopted. As an indication of how much enthusiasm is being aroused on the matter a generous offer towards the cost of building a special cancer ward has been received; and the problem to be faced is whether to endeavour to raise the rest of the necessary money, and to see what promises towards meeting the cost of maintenance can be secured, or whether not to take the risk. Local hospital laboratories have proved so advantageous that, were it possible, there should be a future for local radium institutes. A cautious policy has been decided on, and the war, of course, has been put forward in justification of it. We would only wish to point out that the war is a rather doubtful argument, since the real lesson for voluntary institutions arising from its outbreak has been not that charity is all but exhausted by the normal demands of peace, but, in fact, that the purse of charity is, like the widow's cruse, inexhaustible, if only sufficient motive power is provided to loosen its strings.

### LEECHES FROM AUSTRALIA.

With reference to the much discussed scarcity of leeches, it seems to have been overlooked that the new Pharmacopœia recognises a species of leech which is found in the Australasian Colonies. The variety of which the Master of Christ's College, Cambridge, has succeeded in importing a small quantity from India is no doubt suitable as a stop-gap, but it is to be assumed from the fact that this variety (*Limnatis granulosa*) is not recognised in the new Pharmacopœia that the little animals in question are not so appropriate for surgical use as those from Australia. It is difficult to see why in due course a sufficient supply of the Australian leeches should not be procurable, and it is quite possible that a consignment is already on the way. Presumably there is no difficulty in the way of conveying leeches so far from their habitat; if they can be brought from India with their functions unimpaired they can no doubt be brought from Australia. But the dearth of leeches is perhaps not such a serious affair as recent articles in the lay Press would lead the public to suppose. Years ago it would have been difficult to find a chemist's shop without a supply of them, but nowadays they are not often prescribed; in fact, critics affirm that it is not easy to reconcile their use with modern aseptic methods.

### COUNTY COUNCILS AND THE PUBLIC HEALTH.

It was necessarily with a subdued tone that Mr. Herbert Samuel, President of the Local Government Board, addressed last week a meeting of the County Councils Association. He could only point out that the war had profoundly modified all financial plans for the immediate future. The prospect of obtaining the much-needed Exchequer grants in aid of local finance was much less than a few months ago. Nevertheless, he pointed out that the county councils in general respects were extending their usefulness. County councils had been given the chief control in dealing with tuberculosis; and he hoped that delay in making the necessary provision would be now a thing of the past. The Public Health (Prevention and Treatment of Disease) Act, 1913, had enabled the Local Government Board in some counties to make the county council the authority for providing smallpox hospitals. Although the distribution of grants in aid of nursing had been prevented by the war, he would not fail once more to place before the Chancellor of the Exchequer the strong view of the county councils that this work should be entrusted to them rather than to the Insurance Commissioners. If this speech seems cold comfort to the enthusiasts of county council activity, they may be reminded that the pressure of problems created by the war tends to enforce developments and centralisation round these authorities more quickly perhaps than Exchequer grants and fresh legislation. In this respect the institutional outlook is a not unhopeful one, and the schemes which are modified by the war in one respect may be compensated by new practical developments on the other.

### SANATORIUM BENEFIT COMMITTEE.

Mr. J. A. Dawes, M.P., has resigned his position as Chairman of the Sanatorium Benefit Sub-Committee of the London Insurance Committee. Sir Shirley Murphy has been appointed to succeed him, and Mr. R. W. Mottrey has been elected Vice-Chairman.

### THE WAR AND THE SCHOOL MEDICAL SERVICE.

Sir George Newman, in his sixth Annual Report as chief medical officer of the Board of Education, reminds us that the loss of life occasioned by the European war renders the preservation of the health of the rising generation of exceptional importance at the present time. Already, as he points out, "there are some gaps to be filled in before we can expect to get consistent results and permanent improvement in the physical life of the people." These gaps are the periods below school age and between school age and the age of insurance, to say nothing of the threefold training, physical, mental, and technical, of the period of adolescence; while maternity is a problem of itself. With all these last, however, we are not for the moment concerned, but in respect of the period between school age and the age of insurance it is necessary to point out how weak even are our present school service safeguards in the emergency of war. It is reported this week that, owing to a shortage of agricultural labour, the farmers are clamouring for the Education Committees to permit school children, boys and girls, to go to work on the farms. Permission, it is stated, has in several instances been granted, or, which comes to the same thing, it has been decided not to prosecute. With the economic solution of this problem we are not concerned. But we are convinced that, in the interests of the public health, of the great service over which Sir George Newman presides, any reduction of the limitations at present imposed on child labour would be a disastrous mistake. The limitations arose because the necessity for improving the medical and educational efficiency of the child demanded them. At a crisis, therefore, during which the health of the rising generation is our chief asset, to imperil the work already done would be merely to subsidise the farmers at the expense of the national health.

### A WISE DECISION.

Miss M. C. Macdonald, an assistant medical officer in the public health department of the London County Council, has recently notified that body that she has married, but in view of the fact that her husband is on war service, she has asked that she may remain in the service for the present. In the ordinary way Miss Macdonald would be required to resign her appointment, but in the special circumstances it is felt that the operation of the Council's standing order should be suspended so that Miss Macdonald may be retained in the service until the conclusion of the war service of her husband or the expiration of her engagement in March 1917, whichever be the earlier date. The decision is one on which the Council is to be congratulated. We discussed this ban on marriage in The Hospital of April 18, 1914.

### THE BELVIDERE HOSPITAL, GLASGOW, INQUIRY.

Mr. James A. Fleming, K.C., Sheriff of Fife and Kinross, and Professor Harvey Littlejohn, Professor of Forensic Medicine in Edinburgh University, have been appointed by the Local Government Board for Scotland Commissioners to hold an inquiry as to the general treatment of patients in the Belvidere Hospital, Glasgow. The principal facts leading up to the inquiry are as follows. On May 25 last year the Hospitals Sub-Committee received a letter from Mr. Alexander Allan in respect of the treatment of his now deceased child, who was a patient in the Belvidere Hospital in the previous February. The Sub-Committee placed the matter in the hands of a Special Sub-Committee, and as a result of its inquiries the Corporation, on November 26 last, adopted a recommendation by the Health Committee to ask the Local Government Board to hold an inquiry into the general treatment of patients in the hospital. The inquiry will open on February 19.

### A JOURNALIST OF THE OLD SCHOOL.

We much regret to record the death of Mr. Alfred Wilcox, at the age of sixty-five, a colleague on the staff of many years' standing, between whom and many of our readers, it is no exaggeration to say, existed a personal bond of sympathy. He was a striking example of the value of the principles on which the success of the older journalism was built up, and which no "improvements," or changes in the mode by which vastly increased circulations are catered for, alter or diminish in value. The success which his life exemplified proves how superior to brilliance and display are devotion to duty, and care and punctiliousness in the work. He was a born journalist, in the sense that nothing he saw or heard and no one he met, whatever the personality or subject might be, passed through his mind without being examined to see what light or influence either had on hospital and nursing affairs. Almost quixotically unassuming, he possessed an intimate knowledge of many sides of life—for instance, on the diverse subjects of Church affairs, horticulture, and Parliamentary topics his information was very remarkable. Throughout his life he had always been associated with journalism, and as his heart was in his work, nothing that he wrote failed to reveal his sober judgment and stored-up information. A man of principle, if ever there was one, his gentleness of manner, and readiness to enter into other people's point of view, gave him a judgment in the application of principle to every-day nursing affairs which made his advocacy of any cause or defence of any infringed right as statesmanlike as it was unflinching. He leaves behind him a standard of work and a memory of kindness which now makes the sympathy extended to his wife and daughters a living memorial of his abilities and character.

### ORTHOPÆDICS AND THE WOUNDED.

The Alder Hey Hospital, an institution belonging to the West Derby Guardians, has been sanctioned by the War Office for use as an orthopædic hospital for the wounded. About 400 beds are available, a section of which will be reserved for orthopædic cases, which will be in charge of Mr. Robert Jones, the Liverpool surgeon. The patients will be selected from the base hospitals at home and in France, and orthopædics, the value of which has been slowly recognised in this country compared with the position accorded to it on the Continent, is now to be enlisted along with other specialties on behalf of the wounded soldiers. Mr. Robert Jones holds the post of lecturer in orthopædic surgery to the University of Liverpool, and was president of the Orthopædic Section at the International Medical Congress held in London in 1913. Further particulars are given in our "Round the World" article, on page 420.

### CHESTER INFIRMARY'S FINANCES.

A serious financial position has arisen for the Chester Royal Infirmary, a deficit of nearly £2,000 having appeared during the past year owing to decreased receipts and a rise in the expenses. That matters can be improved, however, is clear from the statement of Mr. J. R. Thomson, chairman of the infirmary board, who states that there are only 379 subscribers in the city. The Albert Wood wings, which were opened by the King, have been regularly occupied by sick and wounded soldiers since November 11, and this fact should serve to stimulate the generosity of the city. In fact, as Mr. S. M. Frost, the mayor, in presiding at the annual meeting, remarked, from what the soldiers had told him of the comforts they were receiving, he was sure that the infirmary would not be the loser by having included them among its patients. Indeed, here, we think, should lie the solution of the difficulty caused by the extras to the building fund, amounting to more than the £39,000 subscribed. Can the city glory in the fact that soldiers are being received in the Albert Wood wings while they allow the building debt to rest like an incubus on the institution?

### THE CHADWICK LECTURE.

Dr. F. M. Sandwith's third lecture on "War and Disease," of which a report appears elsewhere in this issue, was listened to by a large number of men, including many members of the Royal Army Medical Corps. Sir Henry Trueman Wood, a former Chadwick Trustee, was in the chair, and after the lecture and its final lantern slides were over, pointed out how the whole resources of science were expended to increase the horrors of war and the agonies of its combatants. These lectures have been remarkable for their historical description of the risks which attend armies from their circumstances, apart altogether from those which scientific weapons cause; and the really tragic feature in the situation is that science provides the means by which men, and indeed civilisations, can be destroyed so much more quickly than they can be restored to health or built up. There is no doubt that such lectures as Dr. Sandwith's provide a perspective for his audience which helps them to appreciate the daily mass of detail concerning army health, treatment of wounds, casualties, and disease. The Chadwick Trustees are certainly to be congratulated on the manner in which they have made use of their opportunity to arrange such a course at the present time.

### ACTIVITIES OF PUBLIC DISPENSERS.

Many of our readers, both in the dispensary and out, will be interested in the appointment of Mr. H. Antcliffe, F.C.S., as President of the South Yorkshire Association of Poor-Law Officers. The new President is principal pharmacist to the Sheffield Guardians, the first of his craft to occupy the chair. He is a man of many interests, being a prominent Freemason, honorary secretary of the Sheffield Pharmaceutical and Chemical Society, and actively interested in National Insurance matters. A year ago we referred to the appointment of Mr. J. Hassall France as President of the Public Pharmacists' Association as a popular appointment. Mr. France has been again elected for the present year. Mr. Montagu Smith, F.C.S., dispenser to the Lewisham Infirmary, to whom we recently referred as joining the Colours, has proceeded to India with his regiment, the Queen's Own Royal West Kents. We wish Sergeant Smith a safe and speedy return. He will be remembered as an always prominent member of the Certified Dispensers' Association in the fight for pharmaceutical recognition.

### HOME-MADE FURNITURE: A GUINEA PRIZE.

In the examples which we gave last week of home-made hospital equipment, we made the following statement which we here commend to the separate attention of our readers. "We should welcome," it was stated at the beginning of the article, "a Hospital Works Department Competition. We offer a prize of one guinea for the suggestion for its organisation decided by the Editor to be the best out of a dozen or upwards." The condition, it will be noted, throws on the inventiveness of the reader the scope of the Competition, and therefore we do not propose to give hints, since there are a variety of ways of making the Competition useful; and its essence lies in the consideration of the best out of several different points of view. The prize of one guinea offered by the Editor for the suggestion which seems to him to be the best should stimulate effort, and will be earned by the reader who best grasps, in our judgment, how a Hospital Works Department Competition could be most usefully organised.

### ARMY DISPENSING AGAIN DISAPPOINTING.

Although from time to time alterations are made in the regulations concerning the appointment of dispensers in the Army, less and less satisfaction is produced. It is hardly necessary that we should reiterate our opinions upon the matter, but the last R.A.M.C. Order of January 21 impels us almost to the belief that the authorities are adamant to a sense of justice. The Order states that the names are wanted of those qualified as follows: Holders of the Apothecaries' Hall certificate, unqualified medical men, and assistants to doctors or dispensers. The pharmaceutical qualification is apparently unknown, although the sister Service not only demands it for its naval hospitals, but honours its holders in the Navy List as "Esquires"! Even the Prison Service requires pharmacists in its convict prisons. We still believe that a common-sense view must penetrate the authorities, and look forward to the day when a Pharmaceutical Service will co-operate with the R.A.M.C.

### GIFTS OF FOOD FROM THE COLONIES.

A number of London hospitals are experiencing great benefit from the generosity of our Colonies in the matter of foodstuffs. New South Wales has sent over vast quantities of food, and the Sydney Consignments Committee, acting through the London Chamber of Commerce, has distributed this throughout the Metropolis, a considerable proportion going to the hospitals. The foodstuffs comprise quarters of beef, carcases of mutton, cases of rabbits, sacks of flour, and boxes of butter. Queensland, another benefactor, has sent large quantities of flour, pressed beef, condensed milk, pineapple, and golden syrup, which have been distributed by the Agent-General in London, Sir Thomas Robinson. Another frequent giver has been the Jamaica Growers' Association, which has sent to various hospitals a large number of boxes of oranges and limes. All these gifts are entirely free of cost to the institutions concerned, and nothing is asked from them but (in some cases) to make their own arrangements for delivery, as, owing to the state of congestion at the docks, carriage is a somewhat difficult problem. The sick poor and wounded soldiers who have benefited thereby owe a great debt of gratitude to our Colonial kinsfolk.

### THIS WEEK'S DRUG MARKET.

The drug market continues active, and the volume of business transacted is larger than at normal times. This, of course, is largely due to the demand emanating from the medical services of our own Army and the Armies of our Allies; business for home consumption is also quite good. Generally speaking, the tendency of prices is in an upward direction. Salicylates are getting still scarcer, and it seems probable that prices will further advance; the supplies from neutral countries, supplemented by those of home manufacture, are at present insufficient to meet the demand in full. Hexamine is rather dearer again. The price of carbolic acid is well maintained. Cascara sagrada has an upward tendency in price. An advance in the price of Epsom salts is expected as a result of difficulty in obtaining raw material. Very high prices continue to be asked for juniper berries, and consequently for juniper oil. Oil of wintergreeen is dearer. Oil of cloves is a little cheaper. Medicinal liquid paraffin is again dearer and getting still scarcer. Thymol is lower in price. Cod-liver oil is practically unchanged; no definite reports regarding the new fishing are to hand as yet. The demand for morphine continues strong, and the price is well maintained. A further advance in the price of ipecacuanha seems probable, stocks being low.

